# Spectrum of mutations in monogenic diabetes genes identified from high-throughput DNA sequencing of 6888 individuals

**DOI:** 10.1186/s12916-017-0977-3

**Published:** 2017-12-06

**Authors:** Vikas Bansal, Johann Gassenhuber, Tierney Phillips, Glenn Oliveira, Rebecca Harbaugh, Nikki Villarasa, Eric J. Topol, Thomas Seufferlein, Bernhard O. Boehm

**Affiliations:** 1 Department of Pediatrics, University of California San Diego, La Jolla, CA USA; 2 Sanofi-Aventis Germany GmbH, Frankfurt am Main, Germany; 3 Scripps Translational Science Institute and Scripps Health, La Jolla, CA USA; 4 Department of Internal Medicine I, Ulm University Medical Centre, Ulm, Germany; 5 Lee Kong Chian School of Medicine, Nanyang Technological University, Singapore, Singapore; 6 Imperial College London, London, UK

**Keywords:** High-throughput sequencing, Monogenic diabetes, Pathogenic variants, Type 2 diabetes, MODY, DNA pooling, Targeted sequencing

## Abstract

**Background:**

Diagnosis of monogenic as well as atypical forms of diabetes mellitus has important clinical implications for their specific diagnosis, prognosis, and targeted treatment. Single gene mutations that affect beta-cell function represent 1–2% of all cases of diabetes. However, phenotypic heterogeneity and lack of family history of diabetes can limit the diagnosis of monogenic forms of diabetes. Next-generation sequencing technologies provide an excellent opportunity to screen large numbers of individuals with a diagnosis of diabetes for mutations in disease-associated genes.

**Methods:**

We utilized a targeted sequencing approach using the Illumina HiSeq to perform a case-control sequencing study of 22 monogenic diabetes genes in 4016 individuals with type 2 diabetes (including 1346 individuals diagnosed before the age of 40 years) and 2872 controls. We analyzed protein-coding variants identified from the sequence data and compared the frequencies of pathogenic variants (protein-truncating variants and missense variants) between the cases and controls.

**Results:**

A total of 40 individuals with diabetes (1.8% of early onset sub-group and 0.6% of adult onset sub-group) were carriers of known pathogenic missense variants in the *GCK*, *HNF1A*, *HNF4A*, *ABCC8*, and *INS* genes. In addition, heterozygous protein truncating mutations were detected in the *GCK*, *HNF1A*, and *HNF1B* genes in seven individuals with diabetes. Rare missense mutations in the *GCK* gene were significantly over-represented in individuals with diabetes (0.5% carrier frequency) compared to controls (0.035%). One individual with early onset diabetes was homozygous for a rare pathogenic missense variant in the *WFS1* gene but did not have the additional phenotypes associated with Wolfram syndrome.

**Conclusion:**

Targeted sequencing of genes linked with monogenic diabetes can identify disease-relevant mutations in individuals diagnosed with type 2 diabetes not suspected of having monogenic forms of the disease. Our data suggests that *GCK*-MODY frequently masquerades as classical type 2 diabetes. The results confirm that MODY is under-diagnosed, particularly in individuals presenting with early onset diabetes and clinically labeled as type 2 diabetes; thus, sequencing of all monogenic diabetes genes should be routinely considered in such individuals. Genetic information can provide a specific diagnosis, inform disease prognosis and may help to better stratify treatment plans.

**Electronic supplementary material:**

The online version of this article (doi:10.1186/s12916-017-0977-3) contains supplementary material, which is available to authorized users.

## Background

Diabetes mellitus is a heterogeneous disorder characterized by high fasting blood glucose levels or hyperglycemia that results from a combination of both genetic and environmental risk factors. Most individuals with diabetes are classified into type 1 (T1D) and type 2 diabetes (T2D). Compared to T1D, which presents early in life and is primarily an auto-immune disorder, T2D represents approximately 90% of all diabetes and typically manifests later in life. T2D is a complex polygenic disease caused by interactions between multiple genetic and environmental factors. Significant progress has been made in understanding the genetic architecture of T2D over the past 10 years [1]. A number of genome-wide association studies in diverse human populations have identified more than 60 common variants and loci associated with risk for T2D [2]. These studies have also revealed a significant overlap between traits and phenotypes of monogenic diabetes with related “common” T2D as a prototypic complex disease [3–6].

In contrast to T1D and T2D, monogenic diabetes represents a form of non-autoimmune, early onset diabetes that is primarily genetic. Maturity onset diabetes of the young (MODY), first reported in 1974 [7], is an autosomal dominant form of non-insulin dependent diabetes that is typically diagnosed before the age of 25. Using linkage analysis in families with a high prevalence of diabetes, mutations in more than 10 different genes have been shown to cause multiple types of monogenic diabetes, each with different clinical presentation [8, 9]. MODY is estimated to represent 1–2% of diabetes [10]. In addition, mutations in several genes are known to cause neonatal diabetes and rare syndromes such as Wolfram syndrome (WS) [11], which includes diabetes among other phenotypes. Common variants in the monogenic diabetes genes *HNF4A* [12] and *WFS1* [13], and a low-frequency variant in the *HNF1A* gene [14] have also been associated with risk for T2D, highlighting the genetic overlap between monogenic diabetes and T2D.

Subjects suspected of having monogenic diabetes based on age of onset, family history, and additional phenotypes are referred for genetic screening. However, not all individuals with monogenic diabetes fulfill the classical criteria of MODY [7, 9, 10]. In addition, individuals without any family history of diabetes are likely to be misdiagnosed as having T1D or T2D [15, 16]. Further, many rare forms of diabetes share clinical features with T2D and are sometimes misdiagnosed as T2D due to a lack of genetic information and atypical clinical presentation. An accurate molecular diagnosis of monogenic forms of diabetes is important for determining the right treatment as well as genetic counseling for their families [17, 18]. For MODY, genetic diagnosis has important therapeutic implications [9]. Diabetic individuals with mutations in the glucokinase gene (MODY2) often require no treatment and have low prevalence of complications such as retinopathy and neuropathy despite lifelong hypergylcemia [19, 20]. On the other hand, individuals with a mutation in the *ABCC8* or *KCNJ11* genes can be successfully treated with sulfonylureas rather than with insulin therapy [9].

Screening of monogenic diabetes genes in a large cohort with a clinical diagnosis of T2D has the potential to identify subjects with misdiagnosed monogenic diabetes, in particular in subjects with early onset of the disease. In recent years, advances in high-throughput sequencing technologies have made it possible to sequence selected regions of the human genome in large numbers of individuals. These targeted sequencing approaches have been utilized to sequence genes associated with diabetes and obesity [21–23]. In this study, we utilized the Illumina high-throughput sequencing technology to sequence 225 diabetes associated genes, including genes implicated in monogenic forms of diabetes and genes near variants identified in genome-wide association studies for T2D. A total of 4016 individuals diagnosed with T2D, including 1346 individuals with diabetes diagnosed before the age of 40 years, and 2872 controls were sequenced in our study. In this paper, we focus on the analysis of variants in 22 genes (Additional file 1: Table S1) that have been associated with monogenic forms of diabetes. These include the 13 MODY genes (GCK, *HNF1A*, *HNF4A*, *HNF1B*, *INS*, *NEUROD1*, *PDX1*, *PAX4*, *ABCC8*, *KCNJ11*, *KLF11*, *CEL*, and *BLK*), 6 genes associated with recessive diseases that include diabetes as a phenotype (*WFS1*, *NEUROG3*, *EIF2AK3*, *GLIS3*, *RFX6*, and *SLC19A2*), and 3 genes in which heterozygous mutations have been shown to cause diabetes mellitus (*PAX6*, *GATA6*, and *PPARG*). Our primary objectives were to (1) identify subjects with potentially undiagnosed monogenic diabetes, (2) compare and contrast the frequency of deleterious mutations in monogenic diabetes genes between individuals with early-onset diabetes or adult-onset diabetes and population controls, and (3) assess the relationship between deleterious mutations in less frequently mutated monogenic diabetes genes and risk for early onset diabetes.

## Methods

### Cohorts

All samples were obtained through the Centre of Excellence for Metabolic Disorders, Division of Endocrinology and Diabetes, Ulm University Medical Centre. Diabetes was defined as fasting plasma glucose *>* 125 mg/dL or 2 hour glucose *>* 200 mg/dL after an oral glucose tolerance test. Furthermore, individuals with a history of diabetes or undergoing treatment with oral anti-diabetic drugs (primarily metformin and sulfonylureas) or insulin were considered as cases. All subjects studied were of Northern European ancestry. In addition, all diabetes subjects and the controls were tested for the presence of serum autoantibodies, including islet cell autoantibodies, glutamic acid decarboxylase, and islet antigen 2 antibodies, as previously described [24]. Positivity for islet-cell autoantibodies, insulin requirement, and evidence of ketosis at the time of diagnosis were criteria for exclusion. Exclusion criteria were also pregnancy and the presence of any other severe disease. Each study subject was interviewed regarding their family history; history taken included basic clinical information, namely age at diagnosis, sex, treatment (including time to insulin treatment), body mass index, current glycated hemoglobin (HbA1c), current age, and the presence or absence of a parent with diabetes [25]. No interview or biochemical test was performed in any of the relatives. All subjects diagnosed before 25 years of age did not have the MODY phenotype based on classical diagnostic criteria, namely no treatment with insulin for at least 2 years after diagnosis and multi-generational inheritance of diabetes [7]. All individuals with early onset diabetes were also screened for apparent WS based on phenotypes such as optic atrophy, diabetes insipidus, and deafness. Controls had normal fasting glucose (confirmed by HbA1c *<* 6%) and had no evidence of islet autoimmunity.

The first set of sequenced samples included 1880 individuals with T2D and 1840 controls. The mean age at diagnosis of diabetes in cases was 43.4 years, with 734 individuals classified as having early onset diabetes (age at diagnosis *<* 40 years). The second group of sequenced samples included 2136 individuals with T2D (612 individuals with age at diagnosis < 40 years) and 1032 population controls (age *>* 65 years) from the southern part of Germany. For a subset of individuals, additional phenotype information about the presence of diabetic complications (nephropathy, neuropathy, and retinopathy) was also available. Clinical characteristics and phenotype data (age at diagnosis, body mass index, and HbA1c or fasting blood glucose) from the case and control populations are reported in Additional file 1: Table S4.

### Selection of genes for sequencing

Although high throughput sequencing technologies make it possible to sequence human genomes, it is still costly to sequence the entire human genomes of thousands of individuals. However, targeted sequencing of specific regions (e.g., exons of genes of interest) is feasible in thousands of individuals using the same sequencing throughput. We performed targeted sequencing of the exons and the 5’ and 3’ un-translated regions of genes that (1) are associated with monogenic or rare forms of diabetes, (2) are located near common variants associated with risk for T2D [6], (3) have been linked to diabetes in model organisms, or (4) have relevance for drugs used to treat diabetes. In total, a total of 225 genes were selected for sequencing based on these criteria (Additional file 1: Table S2).

### Target enrichment and pooled sequencing

For enrichment of the targeted regions, we utilized the Agilent SureSelect solution hybridization method [26]. For each gene, exon coordinates were obtained from the RefSeq database to identify the coding and untranslated regions. Subsequently, baits were designed (120 bp length, 2× tiling) targeting the DNA sequence of the selected regions. Although targeted sequencing dramatically increases the cost-efficiency of sequencing, there is a significant cost associated with preparing DNA sequencing libraries for each individual sample. Therefore, to reduce the cost of sequencing per sample, DNA from multiple individuals was pooled prior to library preparation and hybridization. We have previously demonstrated that both rare (even singleton mutations present in moderate sized pools) and common mutations can be detected with high sensitivity and specificity from pooled sequence data [27, 28]. A number of studies have utilized pooled sequencing to search for disease risk variants in selected regions of the human genome for a number of diseases, including T1D [29], inflammatory bowel disease [30, 31], Crohn’s disease [32], anorexia nervosa [33], and breast cancer [34]. Similar to previous studies, the number of individuals in a pool was chosen to be small (20–24) since this significantly reduces the cost of library preparation per individual (8–10 times more individuals can be sequenced for the same cost [27, 28]) but still allows for the accurate detection of variants.

### Study design

**Fig. 1:**
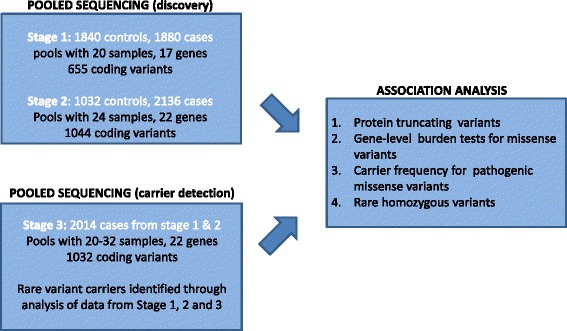
Overview of the sequencing study. A total of 2872 controls and 4016 cases (1346 individuals with age of onset < 40 years) for type 2 diabetes were sequenced using pools of 20 (Stage 1) and 24 (Stage 2) individuals. To validate rare functional variants and to identify the carriers of rare coding variants, 2014 cases selected from Stage 1 and 2 were sequenced again in Stage 3. The resulting variant data was analyzed to perform gene-level burden tests and compare the frequency of protein truncating variants and known pathogenic missense variants in monogenic diabetes genes between the case and control groups

Sequencing of the DNA samples was performed in three stages (Fig. 1). In the first stage, selected regions of 136 genes were sequenced in 1880 individuals with T2D and 1840 controls using a pooled sequencing design (Additional file 1: Figure S2). All pools contained DNA from 20 individuals each and were designed to be homogeneous with respect to the presence or absence of T2D as well as additional phenotypes such as the age of onset (for cases) or current age (for controls) and diabetic complications. Subsequently, in Stage 2, an independent set of 2136 cases and 1032 controls was sequenced using pools of size 24. The pool size was increased to enable the sequencing of additional samples. Finally, to validate deleterious variants identified in Stages 1 and 2 and to identify the carriers of rare coding variants, we performed pooled sequencing of DNA from 2014 individuals with diabetes (1268 early-onset and 746 late-onset) that were also sequenced in Stage 1 and 2. Pools from Stage 1 and 2 with deleterious variants (e.g., missense mutations in *GCK*) were prioritized for sequencing in Stage 3. The pools in Stage 3 were designed to be orthogonal to pools in Stages 1 and 2 such that a pool from the first two stages of sequencing and a pool from the third stage shared at most 1–2 individuals (Additional file 1: Figure S2).

### Library preparation and sequencing

For each individual, DNA was quantified in duplicate (or triplicate if necessary) using PicoGreen. Subsequently, samples were pooled in equimolar concentrations to form pools with DNA from the selected number of individuals. The pools were then carried through the standard Illumina library preparation process using Adaptive Focused Acoustics for shearing (Covaris), end-repair, A-tailing, and ligation. Agilent SureSelect in-solution hybridization was performed on the pooled samples using the recommended protocol for a single genomic DNA sample as previously described [28]. Captured DNA was then sequenced using a 100 bp paired-end multiplexed read protocol on an Illumina HiSeq instrument.

### Read alignment and variant calling

The paired-end reads for each pool were aligned to the human genome reference sequence (hg19) using the Novoalign alignment program [35] (with soft-clipping (v3.0) to generate a BAM file. The BAM file was sorted and PCR duplicates were removed using the Picard [36] MarkDuplicates command. Subsequently, the bam files for the pools were processed using the CRISP variant calling program [27] to identify variants (details in Additional file 2: Supplementary Methods). Variants were identified for pooled sequence data for each stage separately but jointly across all pools. Variant calls were restricted to the targeted regions and the 100 base pairs flanking the targeted regions.

### Variant annotation

All identified variants were annotated using the Annovar annotation program using the RefSeq transcript database [37]. We focused primarily on coding variants that are predicted to impact the protein sequence, namely (1) missense variants (including non-frameshift insertions or deletions (indels)) and (2) protein truncating variants (nonsense, splice-site and frameshift indels). Missense variants were further annotated using the in silico prediction tools PolyPhen2 [38], SIFT [39], MutationTaster [40], and CADD [41]. Alignments for protein truncating variants were inspected visually and variants with weak read support were removed. We utilized variant calls and allele frequency data from the National Heart, Lung, and Blood Institute Exome Sequencing Project [42] and the Exome Aggregation Consortium (ExAC) database [43] to estimate the allele frequencies of the variants. Information about missense mutations that have been reported to be associated with early onset diabetes and MODY was obtained from published papers and the Human Gene Mutation Database [44]. Variants that have been shown to not impact gene function or with a high allele frequency in controls were not considered as pathogenic. Rare variants were further classified using a five-tier classification system as per the American College of Medical Genetics (ACMG) guidelines [45]. Each variant was classified as ‘Benign’ (class 1), ‘Likely benign’ (class 2), ‘Unknown significance’ (class 3), ‘Likely pathogenic’ (class 4), and ‘Pathogenic’ (class 5) using the bioinformatics tool InterVar [46], ClinVar [47] and clinical, functional and genotype-phenotype data from the literature.

## Results

### Description of variants identified and data quality

In the first stage, targeted sequencing was performed on 1880 individuals with diabetes and 1840 controls using 186 pools. Analysis of the read depth across the coding sequence of the sequenced genes showed that the median coverage per pool varied from 600× to 970× per base. The fraction of the targeted bases with a read depth of 200× or greater (10× per individual in a pool with 20 individuals) varied between 0.79 and 0.87 across the pools and was slightly higher in the control pools (0.84 ± 0.02) compared to cases (0.834 ± 0.02) (see Additional file 1: Figure S3 for a distribution of coverage across pools). A small number of targeted exons had a low read depth across all sequenced pools in Stage 1 as well in Stage 2 (Additional file 1: Table S8); 5/7 of these exons also had low sequence coverage (<10× median coverage) in large-scale exome sequence datasets and 3 of these exons correspond to GC-rich regions (GC% ≥ 70%, Additional file 1: Table S8). Excluding these 7 exons with low read depth, 88.7% of the targeted bases were well covered at a threshold of 200×. Further, using a stringent coverage criteria (≥ 90% pools with ≥ 200× coverage at each base), 79% of the targeted bases in the 17 monogenic diabetes genes (~25 kilobases of DNA sequence) were well covered. For two genes, *PDX1* and *INS*, less than 40% of the bases were well covered. Both of these genes also had low sequence coverage in Stage 2 pools (Additional file 1, Table S1) and were difficult to sequence using target capture-based methods [22].

Analysis of the sequence data for the 186 pools using a pooled variant calling method, CRISP [27], identified 655 coding variants in 17 monogenic diabetes genes that included 253 (38.6%) synonymous single nucleotide variants (SNVs), 379 missense SNVs, 3 stop-gain mutations, and 18 indel variants (Additional file 1: Table S3). Most of the detected variants were very rare, with 54% of the variants having an estimated allele count of 1 (also known as singletons) and 81% of the variants estimated to have an allele frequency of 0.001 or lower (Additional file 1: Figure S1). To assess the sensitivity and specificity of variant detection from pooled sequencing, we sequenced 20 samples from one pool individually using the same target capture and library preparation protocols. Overall, 47 variants were identified from the analysis of individual-level sequence data of the 20 samples, while 45 variants were detected from the pooled data, 44 variants overlapped, and 2/3 variants unique to the individual sequence data had low coverage in the pooled data (2–3× per individual). From this data, we estimated a low rate of false positive variants per pool (< 3%) and a low false negative rate (< 7%) primarily due to low sequence coverage.

To assess the accuracy of the variants identified from the pooled sequence data, we compared the variants and their allele frequencies with exome sequence data from the National Heart, Lung, and Blood Institute Exome Sequencing Project [42]. This comparison demonstrated a high sensitivity for the detection of even low frequency variants (minor allele frequency ≥ 0.001) and high concordance of variant allele frequencies (*r* ^2^ = 0.998 for all SNVs, Additional file 2: Supplementary Methods). Furthermore, using Sequenom genotyping of 23 SNVs in 240 individuals, the allele counts at individual variant sites estimated from the pooled sequence data were observed to be highly accurate (*r* ^2^ = 0.998, see Additional file 2 for details).

In the second stage of the study, targeted sequencing was performed on DNA from 3168 individuals using 132 pools (43 control pools and 89 case pools with 24 individuals per pool). Of the 1044 variants detected, 602 (56.7%) were missense variants and 18 were insertion/deletion variants. The fraction of missense variants was very similar to the fraction of missense variants (0.6) in the first stage of the sequencing. The number of variants detected was greater than in Stage 1 since five additional monogenic diabetes genes (*CEL*, *EIF2AK3*, *ABCC8*, *BLK*, and *KLF11*) were sequenced (Additional file 1: Table S1). Overall, 88.9 ± 1.3% of the targeted bases across the 22 genes (38 kilobases of DNA sequence) had a median read depth of ≥ 240× (10× per individual in pools of size 24). The coverage was slightly higher in the control pools compared to the case pools (Additional file 1: Figure S3). The allele frequency distribution of rare variants (Additional file 1: Figure S1) and the proportion of singleton variants was similar to that observed in the first stage of sequencing.

In Stage 3, 1011 coding variants were identified from the sequencing of 2014 individuals with diabetes. These variants included 585 missense SNVs and 21 indel variants (Additional file 1: Table S3). Through joint analysis of pools sequenced in Stage 3 and the corresponding pools in Stages 1 and 2 as well as information about the overlap between pools, we identified the carrier(s) of each rare variant using a parsimonious approach (Additional file 2: Supplementary Methods). There was strong agreement between the observed number of carriers of the variant allele in Stage 3 data and the expected number of carriers from Stage 1 and 2 data. In the *GCK*, *HNF1A*, and *HNF4A* genes, 51 rare missense and protein truncating variants (allele counts < 5) were observed in pools from Stage 3. For 48 of the 51 variants, the expected and observed allele counts were perfectly consistent with data from Stages 1 and 2 and carriers could be identified with little or no ambiguity. Low sequence coverage in Stage 1 and 2 data explained the discrepancy for the three variants. Overall, less than 7% of rare coding variants in the 17 monogenic diabetes genes that were sequenced in all three stages had discrepancy between variant-positive pools, and these were primarily due to sites with low sequence coverage in Stage 1 data compared to Stage 3. The orthogonal pooled sequencing provided independent validation of the sequence variants detected in Stage 1 and 2 data since each individual with the variant allele was sequenced twice in two different pools and library preparation was also performed independently. For variants with multiple variant-positive pools in Stage 3 as well as in Stages 1 or 2, there was some ambiguity in identifying variant carriers. Nevertheless, even in such cases, the orthogonal pooled sequencing enabled partial carrier identification and provided useful information about the age of diagnosis of the individual(s) with the variant. Information about variants identified in Stage 3 pools was not used for comparison of variants between cases and controls but only to validate rare variants and identify the carriers (and age of onset) of rare deleterious variants.

### Analysis of protein-truncating variants

Genetic variants that result in a premature stop codon in the transcript are commonly referred to as protein truncating or loss-of-function variants and typically result in a severe impact on gene function. Seven such mutations were observed in MODY genes in which heterozygous loss-of-function variants are known to be pathogenic for diabetes – three each in the *GCK* and *HNF1A* genes and one in the *HNF1B* gene (Table 1). All seven variants were singletons and each of the seven individuals with these mutations had diabetes (mean age at diagnosis = 27.5 years). The individual with the protein truncating variant (PTV) in the *HNF1B* gene was diagnosed at 14 years of age and likely has MODY5. Although the cases were screened for classical MODY phenotypes, some subjects with MODY can only be identified via genetic analysis. A recent study showed that the majority of individuals with early onset diabetes that were found to carry MODY mutations using genetic testing were clinically misdiagnosed [48].

**Table 1:** List of protein truncating variants identified in monogenic diabetes genes in which heterozygous protein truncating variants are known to be pathogenic for diabetes. None of the variants were present in the ExAC database

			Counts				
Gene	DNA change	AA change	Cases	Early onset	Controls	dbSNP 144	ACMG class^a^
*GCK*	c.871A > T	p.K291*	1	1	0	rs193922335	5
*GCK*	c.1340_1368del	p.R447fs	1	1	0	─	4
*GCK*	c.863 + 1G > T	p.?	1	0	0	─	4
*HNF1A*	c.994delG	p.E332fs	1	1	0	─	4
*HNF1A*	c.955 + 1G > T	p.?	1	1	0	─	4
*HNF1A*	c.1730_1733dupACCT	p.Q579fs	1	0	0	─	4
*HNF1B*	c.1005dupC	p.H336fs	1	1	0	─	4
*PPARG*	c.465delC	p.H155fs	1	0	0	─	4

Reference sequences: *GCK*, NM_000162; *HNF1A*, NM_000545; *HNF1B*, NM_000458; *PPARG*, NM_005037

^a^ACMG classification: 5 = pathogenic, 4 = likely pathogenic, and 3 = uncertain significance

*AA* amino acid, *ACMG* American College of Medical Genetics, *dbSNP* Single Nucleotide Polymorphism Database

Compared to *GCK*, *HNF1A*, *HNF4A*, and *HNF1B*, protein truncating mutations in the other MODY genes are less frequent causes of MODY [49]. For some of these genes, only a few mutations linked to early onset diabetes have been reported. In the *PDX1* gene, a frameshift insertion was detected in a diabetic individual diagnosed at the age of 48 years. Recently, a study from Iceland [50] identified a rare frameshift variant in *PDX1* to be associated with an increased risk of T2D (odds ratio (OR), 2.47). Similarly, the individual with the *PAX4* PTV had adult onset diabetes (age at diagnosis 56 years). In the *KLF11* gene, two PTVs (one in an early onset diabetic patient and the second in a control individual) were detected (Additional file 1: Table S5). No such variants were detected in the *INS*, *BLK*, *NEUROD1*, and *KCNJ11* genes.

The *CEL* gene was sequenced in a subset of the samples and four frame-shift mutations were observed (four carriers in 2136 cases and one carrier in 1032 controls; OR, 1.94; Additional file 1: Table S5). Only one of the four mutation carriers was from the sub-group with early onset diabetes, indicating that, unlike classical MODY genes, heterozygous protein truncating mutations that affect the *CEL* gene are unlikely to be a strong risk factor for early onset diabetes. This is not very surprising since both of the two single base deletions that have been reported to cause *CEL*-MODY impact a VNTR sequence located at the C-terminal of the gene [51] and result in a protein sequence that is unlikely to be affected by non-sense mediated decay [52].

In non-MODY genes with an autosomal dominant disease inheritance, we identified one protein truncating mutation in *PPARG* in an individual diagnosed with diabetes at 41 years of age. The frameshift mutation (deletion of a C) is located in exon 5 (transcript NM_138711) and is predicted to introduce 48 novel amino acids before ending in a premature stop codon. Protein-truncating mutations in *PPARG* are rare but have been described previously in individuals with T2D and partial lipodystrophy [53–55]. In addition, 19 rare PTVs were identified in five recessive monogenic diabetes genes (all carriers were heterozygous) but were not more frequent in individuals with diabetes (0.35% of cases and 0.31% of controls; Additional file 1: Table S5).

### Gene-level association analysis for rare coding variants

To identify associations for rare coding variants with diabetes, we performed gene-level association tests using coding variants (missense and in-frame indel variants with minor allele frequency < 0.2%) detected in each gene (Additional file 2: Supplementary Methods). Association analysis was performed on sequence data from Stages 1 and 2 independently. The *GCK* gene showed a nominal association between rare coding variants and early onset diabetes (*P* = 0.0174 for early onset cases versus controls in Stage 1 and *P* = 0.0013 in Stage 2). Jointly across Stage 1 and 2 data, missense variants in *GCK* (including one in-frame deletion) were detected in 0.5% of cases and 0.035% of controls. Although seven individuals with a *GCK* mutation had early onset diabetes (Table 2), only four of these were diagnosed before 25 years of age. A large number of heterozygous missense mutations that cause *GCK*-MODY have been identified over the years and are distributed across the gene (> 600 mutations were tabulated by Osbak et al. [56]). Comparison to these known mutations revealed that 14 of the 20 missense variants in our cohort have previously been reported in at least one individual with MODY (Table 2). *GCK* has two protein isoforms that differ in the first 15 amino acids and two of the missense mutations were located at positions 10 and 12 in this region. The one missense variant (p.K12R) detected in an individual without diabetes was predicted to be a benign variant in the pancreas-specific splice isoform (Table 2).

**Table 2:** List of missense (and in-frame indels) mutations detected in the *GCK* gene. All mutations (except p.A11T) were observed in a single individual in our dataset

DNA change	AA change	Poly-Phen2^a^	SIFT^b^	MutationTaster^c^	CADD^d^	Age at diagnosis, years	Previously observed in MODY	ExAC AF^e^	dbSNP144	ACMG class^f^
c.484G > A	p.G162S	Pr.D	tol	del	26.5	13	1 family	─	─	3
c.952G > A	p.G318R	Pos.D	del	del	27.2	14	4 families	─	─	4
c.617C > T	p.T206M	Pr.D	del	del	33	19	13 families	─	─	4
c.238G > A	p.G80S	Pr.D	del	del	32	24	2 families	─	rs193922317	4
c.1349C > T	p.A450V	Pr.D	del	del	29.7	27		─	─	3
c.911T > C	p.L304P	Pr.D	tol	del	24.6	28	3 families	─	─	4
c.559G > T	p.D187Y	Pr.D	del	del	33	28	3 families	─	─	4
c.214G > A	p.G72R	Pr.D	del	del	34	29	18 families	─	rs193922289	5
c.118G > A	p.E40K	Pr.D	del	del	33	30	5 families	─	─	4
c.562G > A	p.A188T	Pr.D	del	del	35	30	22 families	0.0001	rs751279776	4
c.640T > G	p.Y214D	Pr.D	del	del	27.2	33		─	─	3
c.131G > A	p.G44D	Pr.D	del	del	29	34	4 families	─	rs193922279	4
c.572G > A	p.R191Q	Pr.D	del	del	35	37	9 families	─	─	4
c.787_801del	p.263_267del	─	─	─	─	39		─	─	4
c.544G > A	p.V182M	Pr.D	del	del	34	41	12 families	─	rs587780345	5
c.706G > A	p.E236K	Pos.D	del	del	33	42	2 families	─	rs587780347	4
c.394G > A	p.D132N	benign	tol	del	23	56	1 family	0.000015	─	3
c.757G > A	p.V253I	benign	tol	del	18.4	61		0.00006	rs748964205	3
c.31G > A	p.A11T	benign	tol	poly	12.8	32, 45		0.024	rs116093166	2
c.35A > G	p.K12R	benign	tol	poly	16.8	NA		0.000015	rs777958777	3

Reference sequence for *GCK*: NM_000162

^a^PolyPhen2 predictions are probably damaging (Pr.D), possibly damaging (Pos.D) and benign

^b^SIFT predictions are deleterious (del) and tolerated (tol)

^c^MutationTaster predictions are disease causing (del) and polymorphism (poly)

^d^CADD scaled C-scores range from 0 to 30. Higher CADD scores correspond to more deleterious variants; a CADD score of 20 (30) corresponds to the top 1% (0.1%) of deleterious substitutions in the human genome

^e^ExAC allele frequency is the maximum allele frequency of the variant allele among the different populations

^f^ACMG classification: 5 = pathogenic, 4 = likely pathogenic and 3 = uncertain significance (see Methods)

*AA* amino acid, *ACMG* American College of Medical Genetics, *AF* allele frequency, *dbSNP* Single Nucleotide Polymorphism Database, *ExAC* Exome Aggregation Consortium, *NA* not available

### Frequency of missense variants in MODY genes

We did not detect a significant association between missense mutations in genes such as *HNF1A* and *HNF4A*, which are frequently mutated in early onset diabetes, likely due to the fact that not all missense mutations in these genes are pathogenic. Previously reported missense mutations in individuals with MODY or early onset diabetes have a strong prior likelihood of being pathogenic. To analyze the frequencies of the carriers of such mutations in our cohort, we analyzed genes (*HNF1A*, *HNF4A*, *HNF1B*, *INS*, *ABCC8*, and *KCNJ11*) in which a significant number of missense mutations have previously been reported in MODY, neonatal diabetes mellitus, or early onset diabetes [57]. We excluded the remaining MODY genes (*CEL*, *PDX1*, *PAX4*, *BLK*, *KLF11*, *NEUROD1*) from this analysis since either very few missense mutations in these genes have been associated with early onset diabetes or the genetic evidence for association is limited. Previously reported mutations that have been shown to be benign using functional assays or have high frequency in controls were also excluded (see Methods).

We identified 23 missense mutations in these six genes that have previously been reported in MODY or early onset diabetes and are likely pathogenic – 14 in *HNF1A*, 3 in *HNF4A*, 5 in *ABCC8*, and 1 in the *INS* gene (Table 3). Overall, 26 of the 29 individuals with these missense mutations had diabetes (OR, 6.24 for cases versus controls; 95% confidence interval 1.9–20.6; Fisher’s exact test *P* = 0.0004), demonstrating that previously reported pathogenic missense mutations in these genes are significantly over-represented in individuals diagnosed with diabetes compared to controls, particularly in the sub-group of individuals with early onset diabetes (OR, 1.99 for early onset versus late onset sub-group). Nevertheless, 50% of mutation carriers with diabetes were diagnosed at 40 years or later, indicating that not all mutations previously reported in individuals with a diagnosis of MODY or early-onset diabetes are fully penetrant. One such mutation, the p.R136W variant (also reported as p.R114W in literature, Table 3), is the most frequently reported *HNF4A* mutation and was detected in two pools consisting of individuals with late onset diabetes (age at diagnosis > 46 years). Recent analysis of this specific mutation has shown that this mutation causes MODY-like diabetes but has lower penetrance in comparison to classical MODY mutations [58]. Mutations that are pathogenic for early onset diabetes are expected to be very rare in the population. Indeed, analysis of the population allele frequencies showed that all variants were very rare and the minor allele frequency for 22 of the 23 variants was less than 0.0005 (Table 3).

**Table 3:** List of missense mutations in the *HNF1A*, *HNF4A*, *HNF1B*, *INS*, and *ABCC8* genes that have previously been reported in individuals or families with MODY or early onset diabetes. The *ABCC8* gene was sequenced in a subset of individuals (2132 cases and 1024 controls)

			Counts										
Gene	cDNA change	AA change	Cases	Early onset	Controls	PolyPhen2^a^	SIFT^b^	MutationTaster^c^	CADD^d^	Previously observed in MODY/diabetes^e^	ExAC AF^f^	dbSNP 144	ACMG class^g^
*HNF1A*	c.391C > T	p.R131W	1	1	0	Pr.D	del	del	31	29 families	─	rs137853244	5
*HNF1A*	c.608G > A	p.R203H	2	1	0	Pos.D	del	del	29	19 individuals	─	rs587780357	4
*HNF1A*	c.812G > A	p.R271Q	1	1	0	Pr.D	del	del	34	13 individuals	0.00007	rs779184183	4
*HNF1A*	c.779C > T	p.T260M	1	1	0	Pr.D	del	del	33	13 families	─	─	4
*HNF1A*	c.1340C > T	p.P447L	1	1	0	Pr.D	del	del	34	11 studies	─	rs137853236	5
*HNF1A*	c.1135C > G	p.P379A	1	1	0	Pr.D	del	del	25	10 studies	0.0006	rs754729248	4
*HNF1A*	c.815G > A	p.R272H	1	0	0	Pr.D	del	del	34	20 families	─	rs137853238	5
*HNF1A*	c.1061C > T	p.T354M	2	1	0	benign	tol	poly	23	3 individuals	0.00006	rs757068809	3
*HNF1A*	c.1513C > A	p.H505N	1	0	0	Pos.D	tol	del	26.1	3 individuals from one study	0.00017	rs577078110	4
*HNF1A*	c.1400C > T	p.P467L	1	0	0	benign	del	del	20.8	3 individuals	0.000015	─	3
*HNF1A*	c.481G > A	p.A161T	0	0	1	Pos.D	del	del	31	1 individual	0.00024	rs201095611	3
*HNF1A*	c.503G > A	p.R168H	0	0	2	Pos.D	del	del	32	1 individual	0.00006	rs377110124	3
*HNF1A*	c.403G > A	p.D135N	1	1	0	Pos.D	del	del	32	1 individual	─	─	3
*HNF1A*	c.1699G > A	p.V567I	1	0	0	benign	tol	poly	18.8	1 individual	0.0001	─	3
*HNF4A*	c.400C > T	p.R134W	1	1	0	Pos.D	del	del	35	5 families	─	rs370239205	4
*HNF4A*	c.406C > T	p.R136W	2	0	0	Pos.D	del	del	34	36 families	0.0001	rs137853336	5
*HNF4A*	c.929G > A	p.R310Q	2	0	0	Pr.D	tol	del	24.7	1 family/co-segregation with diabetes [80]	0.00003	rs371124358	4
*ABCC8*	c.886G > A	p.G296R	1	1	0	benign	del	del	27.1	Individual with diabetes at 7 months [82]	0.00006	rs148529020	3
*ABCC8*	c.1067A > G	p.Y356C	1	0	0	Pr.D	del	del	26.1	Early onset diabetes family [78]	0.00005	rs59852838	4
*ABCC8*	c.2473C > T	p.R825W	2	1	0	Pr.D	del	del	35	Multiple individuals with NDM [83]	0.00001	rs779736828	4
*ABCC8*	c.4136G > A	p.R1379H	1	1	0	Pr.D	del	del	34	One individual with transient NDM [81]	─	─	3
*ABCC8*	c.4516G > A	p.E1506K	1	1	0	Pr.D	del	del	35	Finnish family [77]	─	rs137852671	5
*INS*	c.16C > T	p.R6C	1	0	0	─	del	del	22.7	Three-generation MODY family [76]	0.00006	rs121908278	5

Reference sequences: *HNF1A*, NM_000545; *HNF4A*, NM_000457; *ABCC8*, NM_000352; *INS*, NM_001185098

^a^PolyPhen predictions are probably damaging (Pr.D), possibly damaging (Pos.D) and benign

^b^SIFT predictions are deleterious (del) and tolerated (tol)

^c^MutationTaster predictions are disease causing (del) and polymorphism (poly)

^d^CADD scaled C-scores range from 0-30. Higher CADD scores correspond to more deleterious variants; a CADD score of 20 (30) corresponds to the top 1% (0.1%) of deleterious substitutions in the human genome

^e^Information about previously observed MODY mutations in the HNF1A and HNF4A genes was obtained from Colclough et al. [79]

^f^ExAC allele frequency is the maximum allele frequency of the variant allele among the different populations reported in the database

^g^ACMG classification: 5 = pathogenic, 4 = likely pathogenic, and 3 = uncertain significance

*AA* amino acid, *ACMG* American College of Medical Genetics, *AF* allele frequency, *dbSNP* Single Nucleotide Polymorphism Database, *ExAC* Exome Aggregation Consortium, *NA* not available, *NDM* neonatal diabetes mellitus

Combined with the 14 missense mutations in the *GCK* gene, the overall frequency of previously reported pathogenic missense mutations was 1.8% in early onset diabetes (24/1346), 0.6% in late onset cases (16/2670), and 0.1% in controls (Additional file 1: Table S7). Overall, analysis of rare missense mutations in these genes indicated that previously reported pathogenic missense mutations were significantly over-represented in individuals with diabetes compared to controls (OR, 9.3; *P* = 5 × 10^–7^). Analysis of rare missense variants classified as likely pathogenic or pathogenic (class 4 or 5) using the ACMG guidelines [45] showed a similar trend, wherein 1.5% of individuals in the early onset diabetes sub-group, 0.4% of individuals in the late onset sub-group, and none of the controls carried such mutations in the *GCK*, *HNF1A*, *HNF4A*, *ABCC8*, and *INS* genes (Tables 2 and 3).

The detection of a significant number of individuals with previously reported pathogenic missense variants indicated that additional, previously unreported pathogenic mutations could also be present in the data. Therefore, we analyzed missense mutations that are predicted to be deleterious by the two leading in silico annotation tools (Polyphen2 and SIFT) and have low population allele frequency (minor allele frequency < 0.0005). All of these missense variants were also classified as deleterious by MutationTaster and CADD (C-scores > 20); 18 such missense mutations were observed in the sequence data with 18 carriers in cases and 6 in controls (OR, 2.15; Additional file 1: Table S6), indicating that additional pathogenic mutations likely exist in the sequenced data but are difficult to pinpoint without functional or genetic data.

### Variants in recessive monogenic diabetes genes

Next, using information about the carriers of rare variants identified from Stage 3 sequence data, we searched for individuals who were homozygous for rare coding mutations in six recessive monogenic diabetes genes. We identified an individual who is likely homozygous for a rare missense variant (NM_001145853; exon 8; c.1672C > T; p.R558C) in the *WFS1* gene. Homozygous or compound heterozygous mutations in *WFS1* cause WS, which is characterized by a lack of insulin secretion leading to diabetes mellitus, optic atrophy, and several other phenotypes [11]. This individual was diagnosed with diabetes at the age of 14 years but does not have additional symptoms typically associated with WS such as diabetes insipidus, deafness, optic atrophy, or renal and neurological problems. This same variant has previously been reported in two individuals with WS, namely in an individual with an atypical presentation of the disease who was identified to be a homozygous carrier for this variant [59] and in another individual who carried this variant in combination with a coding deletion variant [60]. The second individual had a mild phenotype with diabetes and optic atrophy without other phenotypes of WS. The p.R558C variant is a rare variant with an allele frequency of 0.0008 in individuals of European ancestry from the ExAC database [43] and even lower in other populations. In our dataset, the frequency of this variant was 0.0007, similar to that observed in the ExAC database.

## Discussion

In this study, we sequenced and analyzed mutations in monogenic diabetes genes in a large cohort of individuals with diabetes (*n* = 4016) and controls (*n* = 2872) from the southern part of Germany. Among individuals with young onset and adult onset diabetes, 40 individuals (1.8% of subjects with early onset diabetes and 0.6% with late onset) were carriers of known pathogenic missense mutations in the *GCK*, *HNF1A*, *HNF4A*, *HNF1B*, *ABCC8*, and *INS* genes. Additionally, protein truncating mutations in these genes were identified in seven individuals with diabetes. The diabetes phenotype of these individuals is likely “*dominated by perturbation in a small number of processes*” related to islet-cell function and hence their diagnosis and treatment can benefit from this knowledge [61]. Although pathogenic missense and PTVs in these genes were strongly enriched in individuals with early onset diabetes, none of these participants fulfilled classical Tattersall criteria of monogenic diabetes mellitus. To enable clinicians to discriminate between T2D and MODY, guidelines for selecting individuals for genetic testing based on clinical criteria have been established [25]. Our population study was not designed to include family members and to genotype or phenotype family members, which may have potentially limited the ability to identify individuals with MODY. Nevertheless, several studies have shown that clinical criteria alone are not sufficient to diagnose MODY and genetic testing is needed for a definitive diagnosis [49].

The most commonly mutated genes in MODY are *HNF1A* and *GCK*, followed by *HNF4A* and *HNF1B* [49]. In our data, the maximum number of pathogenic mutations was observed in the *GCK* gene (17 carriers with 14 in the early onset sub-group) followed by *HNF1A. GCK*-MODY is characterized by mild hyperglycemia typically without diabetes associated microvascular and macrovascular complications [20]. Therefore, *GCK*-MODY is perhaps the most likely form of MODY to be misdiagnosed as T2D [20]. Detection of a *GCK* mutation in an individual with T2D is important from a clinical perspective since no medications are necessary for such individuals except for females during pregnancy.

In addition, the frequency of missense pathogenic mutations in commonly mutated MODY genes observed in our cohort was much higher than the frequency of protein truncating mutations, likely due to the fact that individuals with early onset diabetes were screened for MODY using phenotypic criteria and, as a result, the cohort is depleted of individuals with protein truncating mutations in MODY genes likely to be fully penetrant. Approximately 0.6% of individuals with late onset diabetes were observed to be carriers of likely pathogenic missense mutations that have previously been associated in MODY or early onset diabetes, suggesting that individuals with late onset T2D can also harbor deleterious variants in monogenic diabetes genes with moderate penetrance. A recent study by Flannick et al. [22] sequenced seven MODY genes in two large population cohorts and found 0.5–1.5% of individuals to be carriers of rare missense mutations predicted to be deleterious by bioinformatics tools or previously reported in MODY. However, the majority of these individuals were found not to have diabetes. In contrast, our study was a case–control study and included a large number of individuals (1346) with early onset diabetes. Rare missense mutations that have been previously associated with MODY or early onset diabetes were strongly enriched in the sub-group with early onset diabetes. Another recent large-scale exome sequencing study found a modest but statistically significant enrichment of rare deleterious variants in monogenic diabetes genes in individuals with T2D compared to controls [62].

We detected multiple early onset diabetes subjects with pathogenic missense mutations in the *ABCC8* gene. Such individuals can be treated effectively with sulfonylureas rather than insulin or other medications. The *ABCC8* gene is considered for genetic testing in neonatal diabetes but several studies have identified *ABCC8* missense mutations in individuals with early and late onset diabetes [63–65]. In addition, one individual with early onset diabetes was homozygous for a rare and pathogenic missense variant in the *WFS1* gene, suggesting that genetic testing can identify individuals with an atypical presentation of WS.

Although sequencing can identify pathogenic mutations in genes strongly linked with disease, such as MODY genes, it is challenging to distinguish such mutations from the vast number of neutral mutations observed in large-scale sequencing studies [66]. In the *GCK* gene, our data suggests that the vast majority of missense mutations increase the risk for diabetes to a varying degree. However, in MODY genes such as *HNF1A* and *HNF4A*, not all missense mutations increase the risk for diabetes and, therefore, it is challenging to ascribe pathogenicity to a novel missense mutation based on predictions made by bioinformatics tools. In our data, we did not observe a significant association between rare missense mutations in the genes predicted to be deleterious by multiple bioinformatics tools and risk of diabetes. Recently, Najmi et al. [67] used functional assays to evaluate the missense mutations in the *HNF1A* gene identified by Flannick et al. [22], and showed that 11 of these mutations that reduced transcriptional activity were strongly associated with an increased risk of diabetes (OR, 5.04). Functional assays have been used to identify pathogenic variants in other genes linked with diabetes [68, 69]. Therefore, classification of novel missense variants identified in our study using functional assays has the potential to identify additional individuals with mutations that increase the risk of MODY or T2D.

Our study leveraged the massive throughput of high-throughput sequencing instruments and the ability to sequence selected regions of the human genome in large numbers of individuals. We utilized a pooled DNA sequencing approach to reduce the cost of DNA library preparation. Although pooled sequencing was highly cost-effective and allowed us to sequence nearly 6900 individuals with high sensitivity and specificity for the detection of rare variants, it is less informative than individual sequencing about individual genotypes and does not allow for the detection of copy number variants such as large deletions. In addition, some of the genes targeted for sequencing in our study had low sequence coverage (e.g., the *INS* gene) and we estimated a false negative rate of ~7% for the discovery of rare variants. As a result, a small number of pathogenic mutations were likely not detected. It is possible that additional pathogenic variants (e.g., in non-coding regions) in known monogenic diabetes genes as well as novel genes for early onset diabetes remain to be identified, thereby defining new variants with a large effect on the disease phenotype. Another limitation of our study is the lack of family data or access to DNA samples from first degree relatives of individuals with diabetes for further genotype and phenotype studies.

Our cohort represents a relatively homogeneous cohort of European ancestry from the southern region of Germany with well-defined criteria for classifying individuals as cases and controls. All subjects had been screened for the presence of islet cell autoimmunity to exclude the presence of classical autoimmune diabetes (T1D) and late onset/latent autoimmune diabetes in adult [70]. Many large scale studies of the genetics of T2D do not measure islet cell antibodies and, therefore, exclude subjects with an early age of onset to avoid including T1D cases. In addition, in view of the high prevalence of subjects with latent autoimmune diabetes in adult onset diabetes subjects a major confounding factor can be present in genetic studies of the so-called T2D [71]. There is growing evidence from genetic studies for the heterogeneity of the adult onset diabetes phenotype and overlap with monogenic diabetes [67] and T1D [70]. Multiple studies have shown that 5–15% of individuals with so called T2D are positive for islet cell antibodies [72, 73]. Recent work has addressed the question of a fine-grained categorization of adult onset diabetes using clinical data in large cohorts [74].

## Conclusion

In our sequencing study involving 6888 individuals, 2.2% of individuals with early onset diabetes and 0.7% of individuals with late onset diabetes harbored a likely pathogenic mutation in monogenic diabetes genes. Our results confirm previous reports that MODY is under-diagnosed [19, 75], particularly in individuals presenting with early onset diabetes and clinically labeled as T2D and, in such cases, genetic testing can provide an etiological diagnosis. With the continuing reduction in costs of DNA sequencing, genetic screening of all known monogenic diabetes genes in individuals with early onset diabetes should be routinely considered since it can identify individuals with undiagnosed MODY as well as atypical forms of monogenic diabetes. Knowledge of mutations in monogenic diabetes genes has the potential to influence diagnosis and therapy for individuals with diabetes as well as to enable the genetic testing of relatives.

## Additional files

Additional file 1: Table S1. List of 22 genes associated with monogenic forms of diabetes that were analyzed in this paper. **Table S2.** Criteria used to select genes for targeted sequencing. **Table S3.** Summary of samples sequenced in Stages 1, 2, and 3, and the coding variants identified in each stage. **Table S4.** Clinical data of the cases and controls for type 2 diabetes sequenced in this study. **Table S5.** List of all protein truncating mutations identified in the 22 monogenic diabetes genes. **Table S6.** Rare missense mutations in the *HNF1A*, *HNF4A*, *HNF1B*, *ABCC8*, and *KCNJ11* genes predicted to be deleterious by PolyPhen2, SIFT, and MutationTaster. **Table S7.** Number of individuals with protein truncating variants and previously reported pathogenic missense variants in MODY genes. **Table S8.** List of exons with low sequence coverage in data from Stage 1 and 2 pools. **Figure S1.** Minor allele frequency distribution of variants identified from sequencing of pools in Stages 1 and 2. **Figure S2.** Pooled sequencing design of the study. **Figure S3.** Comparison of sequence coverage between cases and controls. (PDF 700 kb)

Additional file 2: Supplementary Methods: Description of methods for pooled variant calling, gene-level tests for rare coding variants, statistical analyses, comparison of pooled sequence data with population exome data, comparison of pooled allele counts with individual genotypes, and identification of the carriers of rare variants. (PDF 671 kb)

## Abbreviations

ACMG: American College of Medical Genetics
ExAC: Exome Aggregation Consortium
HbA1c: glycated hemoglobin
Indel: insertion/deletion
MODY: Maturity onset diabetes of the young
PTV: protein truncating variant
SNV: Single nucleotide variant
T1D: type 1 diabetes
T2D: type 2 diabetes
WS: Wolfram syndrome
