# Supplementary material for: Spectrum of mutations in monogenic diabetes genes identified from high-throughput DNA sequencing of 6888 individuals
Source: BMC Med. 2017 Dec 6;15:213. doi: 10.1186/s12916-017-0977-3 (PMC5717832; doi:10.1186/s12916-017-0977-3)
Supplement: Supplementary file 1 — List of 22 genes associated with monogenic forms of diabetes that were analyzed in this paper. Table S2. Criteria used to select genes for targeted sequencing. Table S3. Summary of samples sequenced in Stages 1, 2, and 3, and the coding variants identified in each stage. Table S4. Clinical data of the cases and controls for type 2 diabetes sequenced in this study. Table S5. List of all protein truncating mutations identified in the 22 monogenic diabetes genes. Table S6. Rare missense mutations in the HNF1A, HNF4A, HNF1B, ABCC8, and KCNJ11 genes predicted to be deleterious by PolyPhen2, SIFT, and MutationTaster. Table S7. Number of individuals with protein truncating variants and previously reported pathogenic missense variants in MODY genes. Table S8. List of exons with low sequence coverage in data from Stage 1 and 2 pools. Figure S1. Minor allele frequency distribution of variants identified from sequencing of pools in Stages 1 and 2. Figure S2. Pooled sequencing design of the study. Figure S3. Comparison of sequence coverage between cases and controls. (PDF 700 kb) [file 12916_2017_977_MOESM1_ESM.pdf]

SUPPLEMENTARY TABLES AND FIGURES

Table S1: List of 22 genes associated with monogenic forms of diabetes that were analyzed in this paper. For each gene, we calculated the fraction of bases in each gene for which the read depth per base (median across all sequenced pools) was greater than a specific threshold.

gene	associated phenotype(s)	disease model	bases with ≥ 200x median coverage in stage 1 pools	bases with ≥ 240x median coverage in stage 2 pools
HNF4A	MODY1	AD	0.79	0.93
GCK	MODY2<br>permanent NDM	AD<br>AR	0.85	0.9
HNF1A	MODY3	AD	0.75	0.87
PDX1	MODY4, early onset type 2 diabetes<br>NDM and pancreatic agenesis	AD<br>AR	0.3	0.38
HNF1B	MODY5	AD	0.84	0.87
NEUROD1	type 2 diabetes (MODY6)<br>NDM and neurological abnormalities	AD<br>AR	1	1
*KLF11**	early onset diabetes (rare missense mutations)	AD	-	0.97
*CEL**	diabetes with pancreatic & exocrine dysfunction (frameshift deletion in VNTR region)	AD	-	0.73
PAX4	early onset diabetes (missense mutations)	AD	0.89	1
INS	NDM, MODY and early onset diabetes	AD	0.2	0.42
*BLK**	MODY (missense mutations)	AD	-	0.86
KCNJ11	NDM, early onset diabetes (activating mutations)<br>congenital hyperinsulinism (inactivating or LoF mutations)	AD<br>AR/AD	1	1
*ABCC8**	transient or permanent NDM, early onset diabetes, T2D (activating mutations)<br>congenital hyperinsulinism (inactivating or LoF mutations)	AD<br>AR/AD	-	0.92
GATA6	NDM and exocrine pancreatic insufficiency, adult onset diabetes	AD	0.71	0.74
PAX6	aniridia and type 2 diabetes	AD	0.88	0.99
PPARG	lipodystrophy with type 2 diabetes	AD	1	1
SLC19A2	thiamine-responsive megaloblastic anemia syndrome	AR	0.86	0.87
WFS1	Wolfram syndrome	AR	0.87	0.91
*EIF2AK3**	Wolcott-Rallison syndrome	AR	-	0.91
RFX6	Mitchell-Riley syndrome with neonatal diabetes	AR	0.92	0.99
NEUROG3	Permanent NDM and Enteric Anendocrinosis	AR	0.49	0.64
GLIS3	NDM with Congenital Hypothyroidism	AR	0.83	0.85

NDM = neonatal diabetes mellitus; **AD** = autosomal dominant; **AR** = autosomal recessive
* = not sequenced in stage 1 pools

Table S2: Criteria used to select genes for targeted sequencing.

Selection criteria	Stage 1	Stage 2 & 3
monogenic diabetes	11	13
rare syndromes with diabetes	8	11
obesity	2	4
GWAS for type 2 diabetes	60	65
GWAS for fasting glucose	6	8
genes targeted by drugs treating diabetes	10	10
human candidate gene association studies	5	7
candidate genes (model organisms, functional studies, literature search, pathway analysis)	36	104
genes associated with type 1 diabetes	0	9
pancreas-specific transcription factors	5	7
Total number of distinct genes	136	225

Table S3: Summary of samples sequenced in stage 1, 2 and 3 and the coding variants identified in each stage.

	Stage1	Stage2	Stage 3
Number of cases	1880	2164	2014
cases with age of diagnosis <40 years	734	612	1268
Number of controls	1840	1044	0
Pool size for sequencing	20	24	24-32
Number of genes analyzed	17	22	22
Number of variants detected	655	1044	1011
missense SNVs	379	602	585
insertion/deletion variants	15	18	21
variants with MAF < 0.001	555	889	809

Table S4: Clinical data of the cases and controls for type 2 diabetes that were sequenced in this study. All control samples in stage 2 were > 65 years of age and normoglycemic (mean fasting blood glucose = 90 mg/dl, 95% CI: 74-104).

	cases						controls				
	**N**	**% females**	**age (years)**	**age at diagnosis (years)**	**HbA1c (%)[a]**	**BMI (kg/m^2)**	**N**	**% females**	**age (years)**	**HbA1c (%)**	**BMI (kg/m^2)**
stage 1	1880	44.6	53.6 (17.9)	43.3 (17.2)	7.8 (6.2-9.1)	27.3 (25.2-31.2)	1840	53.7	44.2 (11.1)	4.9 (4.2-5.8)	25.6 (20.7-30.5)
stage 2	2136	37.6	51.3 (14.4)	47.1 (13.5)	8.2 (6.6-9.8)	27.8 (23.6-30.5)	1032	NA	NA	NA	26.6 (24.8-28.7)

NA, not available; BMI, body mass index.
For age and age of diagnosis, data are presented as mean (standard deviation). For HbA1c and BMI, data are presented as mean (95% CI).
[a]HbA1c was measured as defined by the IFCC reference system using a commercially available system (Roche Cobas) and EDTA venous whole blood only

Table S5: List of all protein truncating mutations identified in the 22 monogenic diabetes genes.

								counts				
gene	chr	position	ref	alt	annotation	DNA change	AA change	cases	early-onset	controls	ExAC AF	dbSNP 144
RFX6	6	117250016	GC	G	frameshift	c.2494delC	p.P832fs	1	0	0	–	
SLC19A2	1	169446808	TA	T	frameshift	c.391delT	p.Y131fs	0	0	1	–	
SLC19A2	1	169446794	C	CT	frameshift	c.405dupA	p.A136fs	1	0	0	–	
SLC19A2	1	169446957	G	GT	frameshift	c.242dupA	p.Y81*	1	0	0	0.000015	
WFS1	4	6293061	CT	C	frameshift	c.599delT	p.L200fs	1	1	0	–	
WFS1	4	6302394	A	AC	frameshift	c.877dupC	p.Y291fs	0	0	1	–	
WFS1	4	6303145	T	A	stopgain	c.1623T>A	p.C541*	1	0	0	–	
WFS1	4	6303452	GTGCTGTTC	G	frameshift	c.1931_1938del	p.F646fs	2	2	1	–	rs71526457
WFS1	4	6293688	C	T	stopgain	c.676C>T	p.Q226*	1	0	0	–	
WFS1	4	6302752	CTCTG	C	frameshift	c.1231_1234delTCTG	p.V412fs	1	0	0	0.000015	
WFS1	4	6303043	CTA	C	frameshift	c.1522_1523delTA	p.Y508fs	0	0	1	0.000015	
WFS1	4	6303286	G	A	stopgain	c.1764C>A	p.W588*	1	0	0	0.00006	
WFS1	4	6303360	G	A	stopgain	c.1838G>A	p.W613*	0	0	2	0.0001	
WFS1	4	6304163	TTC	T	frameshift	c.2642_2643delTC	p.F881fs	0	0	1	0.00018	
GLIS3	9	4286332	G	A	stopgain	c.94C>T	p.R32*	3	0	2	0.00003	
GLIS3	9	4117871	C	CA	frameshift	c.1606dupT	p.C536fs	1	0	0	–	
EIF2AK3	2	88857333	CTCTG	C	frameshift	c.3268_3271delCAGA	p.Q1090fs	1	0	0	0.0001	
EIF2AK3	2	88857412	G	A	stopgain	c.3193C>T	p.R1065*	1	0	0	–	
EIF2AK3	2	88885381	A	AG	frameshift	c.1628_1629insC	p.L543fs	0	0	1	–	
GCK	7	44186210	T	A	stopgain	c.871A>T	p.K291*	1	1	0	–	rs193922335
GCK	7	44184764	AGGCCACCGCCGAGACCAGGGCCGCGCCCC	A	frameshift	c.1337_1365del	p.R448fs	1	1	0	–	
GCK	7	44187248	C	A	splice donor	c.863+1G>T	p.?	1	0	0	–	
HNF1A	12	121434102	AG	A	frameshift	c.994delG	p.E332fs	1	1	0	–	
HNF1A	12	121432209	G	T	splice donor	c.955+1G>T	p.?	1	1	0	–	
HNF1A	12	121437391	C	CACCT	frameshift	c.1730_1733dupACCT	p.Q579fs	1	0	0	–	
HNF1B	17	36091624	T	TG	frameshift	c.1006dupC	p.H336fs	1	1	0	–	
CEL	9	135941981	C	CG	frameshift	c.619dupG	p.D207fs	1	0	0	0.0001	
CEL	9	135946653	C	CG	frameshift	c.1777dupG	p.A593fs	1	0	1	–	
CEL	9	135947060	CT	C	frameshift	c.2181delT	p.P727fs	1	0	0	0.0015	
CEL	9	135945971	CA	ACTC	frameshift	c.1419_1420AC	p.T474fs	1	1	0	–	
KLF11	2	10188158	C	T	stopgain	c.694C>T	p.Q232*	0	0	1	0.000015	
KLF11	2	10192441	CAG	C	frameshift	c.1347_48delA	p.T449fs	1	1	0	–	
PAX4	7	127254596	G	A	stopgain	c.352C>T	p.R118*	1	0	0	0.00004	
PDX1	13	28498397	C	CGCCTA	frameshift	c.415_428dupT	p.G144fs	1	0	0	–	
ABCC8	11	17434263	G	A	stopgain	c.2506C>T	p.R836*	1	0	0	0.0006	rs72559722
ABCC8	11	17483210	G	A	stopgain	c.742C>T	p.R248*	1	0	0	–	
PPARG	3	12434180	AC	A	frameshift	c.465delC	p.H155fs	1	0	0	–	

Reference sequences: *RFX6* , NM_173560.3; *SLC19A2* , NM_006996.2; *WFS1* , NM_001145853.1; *GLIS3* , NM_001042413.1; *EIF2AK3* , NM_004836.6; *GCK* , NM_00162; *HNF1A* , NM_000545; *HNF1B* , NM_000458; *CEL* , NM_001807; *KLF11* , NM_001177716; *PAX4* , NM_006193; *PDX1* , NM_000209 ; *PPARG* , NM_005037 ; *ABCC8* , NM_000352;

Table S6: Rare missense mutations in the HNF1A, HNF4A, HNF1B, ABCC8 and KCNJ11 genes that are predicted to be deleterious by PolyPhen2, SIFT and MutationTaster.

				counts						
**gene**	**DNA change**	**AA change**	**CADD**	**cases**	**early-onset**	**cont rols**	**Remarks**	**ExAc AF**	**dbSNP 144**	**ACMG class**
ABCC8	c.916C>T	p.R306C	35	1	1	0	R306H reported in NDM	0.00006	rs751228166	3
ABCC8	c.375C>G	p.H125Q	25.6	4	1	0	reported in hyperinsulinism	0.00015	rs60637558	3
ABCC8	c.403C>G	p.L135V	26.3	0	0	1	L135P reported in NDM	0.00006	rs368450282	3
ABCC8	c.1270G>A	p.D424N	34	1	0	0		0.0003	rs577545383	3
ABCC8	c.1576C>T	p.R526C	34	2	0	0	hyperinsulinism	0.00002	rs779736828	3
HNF4A	c.992G>A	p.R331H	31	0	0	1	transactivation domain	0.00002	rs369429452	3
HNF4A	c.928C>T	p.R310W	32	1	1	0	transactivation domain	0.0001	rs768263630	
HNF1A	c.618G>C	p.W206C	28.5	1	1	0	DNA binding domain	–	–	3
HNF1A	c.1610C>T	p.T537M	35	1	0	0		0.00006	rs372624970	3
HNF1A	c.1544C>T	p.T515M	34	1	0	0		0.00003	rs745460046	3
HNF1A	c.961C>T	p.R321C	26.5	0	0	1		0.00006	rs766770471	3
HNF1A	c.1058C>T	p.P353L	33	0	0	1		–		3
HNF1A	c.824A>C	p.E275A	26.5	2	1	0		0.0001	rs199890776	3
HNF1A	c.451G>A	p.G151S	30	1	0	0		–	–	3
KCNJ11	c.991T>C	p.S331P	26.4	1	1	0		–	–	3
KCNJ11	c.526C>T	p.R176C	32	1	0	0	incomplete penetrance (Edghill et al. 2004)	0.0001	rs201264306	3
KCNJ11	c.292G>A	p.G98S	28.6	1	0	0	hyperinsulinism	–	–	3
HNF1B	c.1325T>C	p.M442T	28.2	0	0	2	outside DNA binding domain	–	rs193922482	3

Reference sequences: *HNF1A* , NM_000545; *HNF4A* , NM_000457; *ABCC8* , NM_000352; *INS* , NM_001185098; *KCNJ11* , NM_000525; *HNF1B* , NM_000458.

CADD scaled C-scores range from 0-30. Higher CADD scores correspond to more deleterious variants; a CADD score of 20 (30) corresponds to the top 1% (0.1%) of deleterious substitutions in the human genome.

AA = amino acid; NA = not available; AF = allele frequency

ACMG classification: 5 = Pathogenic, 4 = likely pathogenic and 3 = uncertain significance (see Methods).

Table S7: Number of individuals with protein truncating variants and previously reported pathogenic missense variants in MODY genes. Only genes with at least one PTV or pathogenic missense variant are listed.

	PTVs			pathogenic missense variants			number of rare missense variants
**gene**	**late-onset**	**early-onset**	**controls**	**late-onset**	**early-onset**	**controls**	
HNF4A	0	0	0	4	1	0	33
GCK	1	2	0	3	11	0	19
HNF1A	1	2	0	6	8	3	52
INS	0	0	0	1	0	0	2
HNF1B	0	1	0	0	0	0	22
ABCC8	2	0	0	2	4	0	57
total	4	5	0	16	24	3	185

Table S8: List of exons with low sequence coverage in data from stage 1 and stage 2 pools. Low coverage was defined as < 200x median read depth in stage 1 pools and < 240x median read depth in stage 2 pools. The median read depth was calculated for each exon and for each pool separately.

				median read depth (inter-quartile range)		median read depth in exome data (ExAc)[b]	
**gene**	**exon (chromosome:start-end)[a]**	**bases**	**GC %**	**stage 1 pools**	**stage 2 pools**	**exon**	**gene**
GATA6	18:19756915-19757082	168	68.4	48 (42-55)	93 (81-109)	8	27
HNF1A	12:121416571-121416897	327	65.7	81 (71-91)	226 (205-259)	20	36
HNF1B	17:36060987-36061182	196	63.8	55 (47-64)	95 (83-104)	5	59.6
INS	11:2181081-2181227	147	63.3	78 (58-90)	170 (154-190)	15.5	34
PDX1	13:28494275-28494681	407	73	72 (58-90)	91 (75-104)	2	17.3
SLC19A2	1:169454800-169455004	205	71.7	32 (25-39)	150 (118-181)	5	61
WFS1	4:6279182-6279414	233	70.4	22 (18-26)	92 (78-109)	8	56.3

[a]hg19 coordinates
[b]Information about read depth for exome data in the ExAc database was obtained from ftp://ftp.broadinstitute.org/pub/ExAC_release/release0.3/coverage

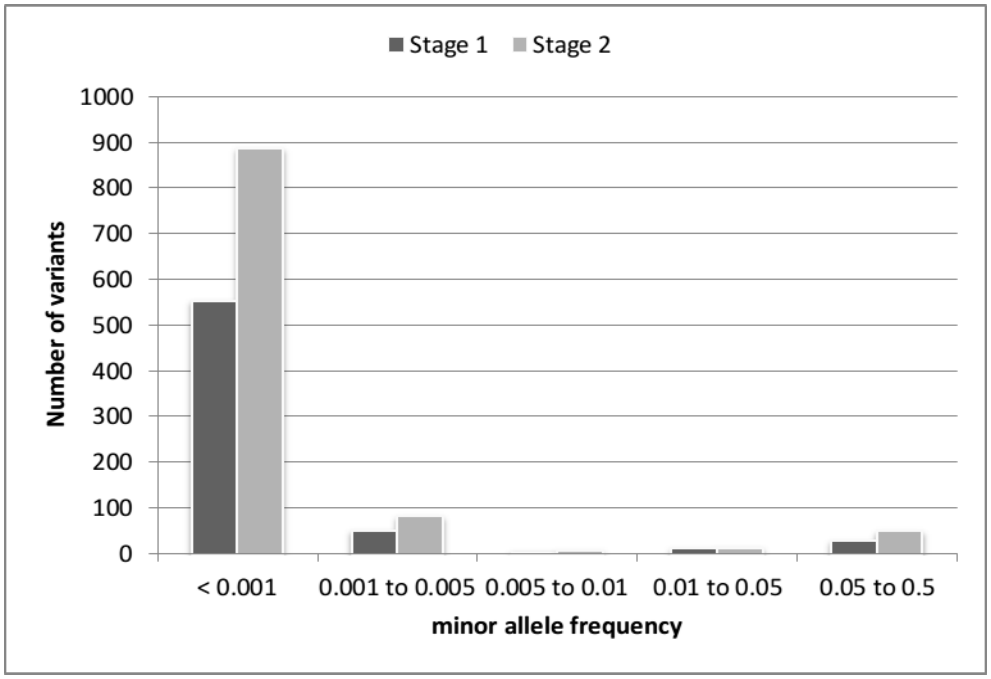

Figure S1: Minor allele frequency distribution of variants identified from pooled sequencing in stage 1 and 2. In stage 1, 84.6% of variants had a MAF less than 0.001. The percentage of such variants in stage 2 was similar at 85.2%.

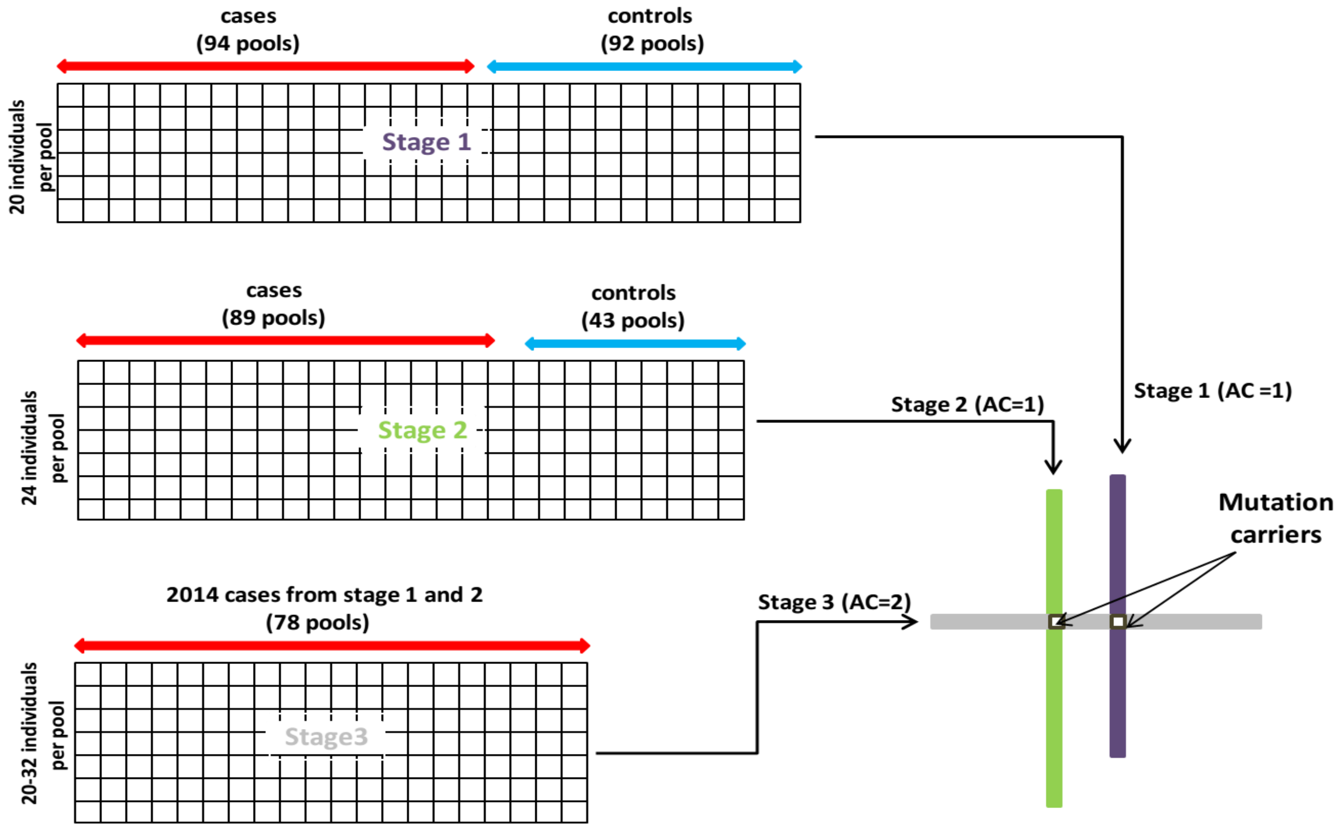

Figure S2: Pooled sequencing design of the study. In stage 1, 1880 cases (94 pools) and 1840 controls (92 pools) were sequenced in pools that consisted of DNA from 20 individuals each. Similarly, in stage 2, 89 case pools and 43 control pools (24 individuals in each pool) were sequenced. In Stage 3, a subset of cases from stage 1 and 2 (early onset and pools with deleterious coding variants) were sequenced using 78 pools that were designed to be orthogonal to pools from stage 1 and 2. Therefore, carriers of rare variants could be identified by using information about the estimated number of carriers in pools and the overlap between pools. As shown in the example, a rare variant with an allele count of 2 (AC =2) in a single pool from stage 3 overlaps with one pool from stage 1 and another pool from stage 2, both of which are variant-positive (estimated allele count of 1). As a result, the two individuals that are heterozygous for the variant allele can be pin-pointed.

Figure S3: Comparison of sequence coverage between cases and controls. (A) Fraction of well covered bases (bases with 200x or greater coverage in each pool) in stage1 between case and control pools. **(B)** Fraction of well covered bases in stage 2. **(C)** Histogram of the fraction of well covered bases per pool (stage 1). Coverage was slightly higher in control pools than case pools (Mann-Whitney rank test, p-value = 0.003 in stage 1 and p-value = 0.04 in stage 2 pools).

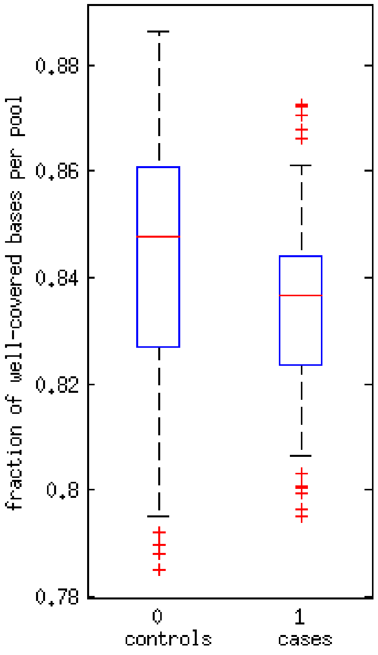

(A)

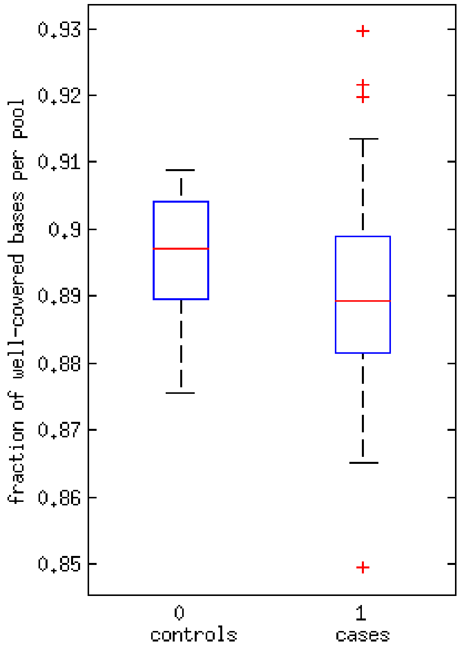

(B)

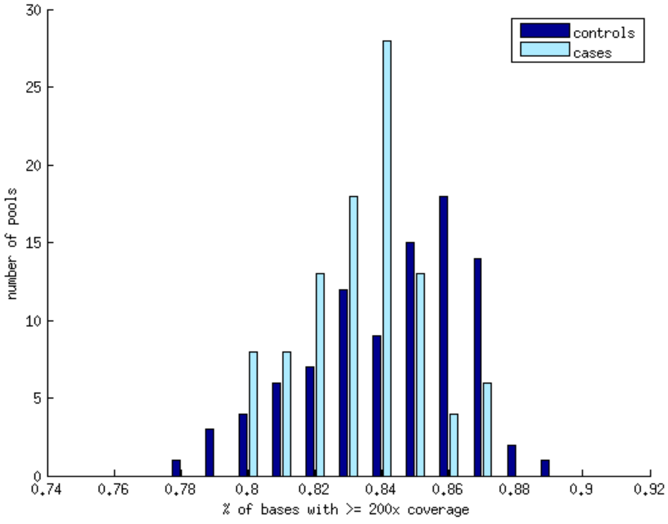

(C)
