# Supplementary material for: Spectrum of mutations in monogenic diabetes genes identified from high-throughput DNA sequencing of 6888 individuals
Source: BMC Med. 2017 Dec 6;15:213. doi: 10.1186/s12916-017-0977-3 (PMC5717832; doi:10.1186/s12916-017-0977-3)
Supplement: Supplementary file 2 — Supplementary Methods: Description of methods for pooled variant calling, gene-level tests for rare coding variants, statistical analyses, comparison of pooled sequence data with population exome data, comparison of pooled allele counts with individual genotypes, and identification of the carriers of rare variants. (PDF 671 kb) [file 12916_2017_977_MOESM2_ESM.pdf]

SUPPLEMENTARY METHODS

Pooled variant calling using CRISP

CRISP uses a two-step approach to identify variants using sequence data from multiple pools which are homogeneous in regards to library preparation and sequencing. First, at each variant site, a statistical method is used to analyze the allele counts across pools to determine the likelihood of the presence of a variant allele in one or more pools. This approach is designed to filter out sites at which systematic sequencing errors or other artifacts can result in false variants. Next, for each potential variant site, CRISP uses an EM algorithm to estimate the likelihood of a variant and the maximum likelihood estimate of the variant allele(s) frequency. CRISP also estimates the number of chromosomes with the variant allele in each pool as follows. We assume that the number of variant alleles can take values from 0 to 2k where 'k' is the number of individuals in the pool. The prior probability for each allele count was calculated using the mean variant allele frequency across all pools estimated by CRISP and the binomial distribution. Using the allele counts and sequencing error rates, the maximum likelihood estimate of the allele count for each pool was calculated and used as the true allele count.

Gene-level tests for rare coding variants

We tested rare variants in each gene collectively for association with type 2 diabetes using two different methods: a simple burden test (59) and the C-alpha test statistic (60). The methods were implemented to work with pooled sequence data and p-values were calculated by permutations of the case-control status. For each gene, the statistical tests were run on two sets of variants: all missense variants and missense variants predicted to be deleterious by PolyPhen. Analysis was done for cases versus controls (stage 1 and stage 2 separately) as well as early onset cases versus controls. We performed the rare variant association tests using a minor allele frequency threshold of 0.002. To avoid false associations due to population stratification, variant allele frequencies from the ExAC database and the 1000 Genomes project were used to exclude rare variants with high allele frequency in non-European populations from the burden tests.

Statistical analyses

We tested for association between phenotype (type 2 diabetes) and variant carrier status (across multiple genes) using Fisher's exact test.

Comparison of pooled sequence data with population exome data

To assess the ability to detect rare variants and estimate allele frequencies from pooled sequence data, we compared the variants identified from the pooled sequence data (Stage 1, 3720 individuals sequenced in 186 pools) and their allele frequencies to exome sequence data from the NHLBI ESP project (4300 exomes of European ancestry). 251 SNVs were shared

between the two variant call sets (limited to the coding regions of 136 genes targeted for sequencing in our study) and all 85 SNVs with a minor allele frequency ≥ 0.001 in the NHLBI exome data were also detected from the pooled sequencing. In addition, no variant that was unique to our dataset had a minor allele frequency greater than 0.0015. This demonstrated that the pooled sequencing was able to detect virtually all variants with a population minor allele frequency (MAF) of 0.001 or greater. In addition, allele frequencies for the variants were highly correlated between the two datasets (r^2 = 0.998 for all SNVs and 0.953 for SNVs with a MAF in the range 0.5-5%) providing another indirect measure of the high quality of our dataset.

Comparison of pooled allele counts to individual genotypes

To assess the accuracy of the allele counts at variant sites from the pooled sequence data, we genotyped 23 SNVs in 240 individuals (from 12 pools sequenced in stage 1) using the Sequenom massARRAY platform. For each SNV, the maximum likelihood estimate of the variant allele count (varying from 0-40) for each pool was compared to the individual genotypes derived allele count. SNV-pool pairs for which one or more individuals in the pool had a missing genotype were not included in the comparison. The allele counts estimated from the pooled sequencing for the 12 pools were highly concordant with the Sequenom genotyping based counts with a Pearson correlation coefficient equal to 0.998. Further, the absolute difference in the allele counts between the pooled sequence data and individual genotype data was never greater than 2 and 64.6% of the SNV-pool pairs were perfectly consistent.

The dbSNP ids of the SNVs that were genotyped were: rs61873492, rs1049846, rs41282898, rs7579712, rs146811884, rs155439, rs11603334, rs3842729, rs2997064, rs3742023, rs2257883, rs2678166, rs74609989, rs60129946, rs75344674, rs73115423, rs141804752, rs1801516, rs78254417, rs6774571, rs7111, rs115663512

Identification of carriers of rare variants

In stage 3, 2014 individuals with diabetes from Stage 1 and 2 were sequenced in 78 pools. The pools in stage 3 were designed to be orthogonal to the pools in stage 1 and 2 such that at most 1-2 individuals were shared between a pool from stage 3 with a pool from stage 1 or stage 2. To identify carriers of rare variants, we used a parsimonious approach that used the information about the individuals present in each pool and the estimated allele counts (per pool) across pools sequenced in Stage 1, 2, and 3. For each variant with at least one variant-positive pool (pool with the variant allele account > 0) in Stage 3 data, the variant-positive pools were intersected with variant positive pools from Stage 1 and 2 to identify potential carriers of the variant allele (see Supp. Figure 2). Variant-positive pools that intersect exactly one another pool enable the identification of carrier without ambiguity. Once the variant carrier(s) in a pool is identified, the particular pool can be removed from the analysis and the procedure can be repeated. Notably, the carrier analysis could also identify individuals that

were homozygous carriers of a rare variant since for such variants; the allele count of the variant positive pool was 2, both in Stage 3 and Stage 1 (or 2).

For each variant, using the estimated allele counts from stage 1 & 2 pools sequenced in stage 3, we can calculate the expected allele count in stage 3 data. Furthermore, since each individual in stage 3 was sequenced in stage 1 or 2, every variant-positive pool in stage 3 should overlap with at least one variant-positive pool from either stage 1 or 2. This provides an estimate of the false negative and false positive rate. Analysis of singleton variants demonstrated that the false positive rate was very low (less than 1%) and the false negative rate was almost completely driven by low sequence coverage in some regions in stage 1 and 2 data. For example, in the *GCK* gene, carriers could be identified for each nonsynonymous or protein truncating variant.
